# SIRT1 in Secretory Organ Cancer

**DOI:** 10.3389/fendo.2018.00569

**Published:** 2018-09-24

**Authors:** Raffaele Frazzi

**Affiliations:** Laboratory of Translational Research, Azienda Unità Sanitaria Locale - IRCCS di Reggio Emilia, Reggio Emilia, Italy

**Keywords:** SIRT1, cancer, secretory organs, acetylation, epigenetic modulation

## Abstract

Mammalian silent information regulator 1 (SIRT1) is reported to play a role in cancers of the secretory organs, including thyroid, pancreatic endocrine, and ovarian tumors [1, 2, 3, 4]. A recent meta-analysis conducted on 37 selected studies of human cancers analyzed the correlations of overall survival (OS), disease-free survival (DFS) and relapse-free survival (RFS) with SIRT1 expression [5]. This study reported that SIRT1 overexpression was associated with a worse OS in liver and lung cancers, while it was not correlated with OS in breast cancer, colorectal cancer, or gastric carcinoma. Collectively, the meta-analysis revealed that an unfavorable OS was associated with SIRT1 expression for solid malignancies. Given the growing importance of this class of lysine/histone deacetylases in human endocrine malignancies, a rational and focused literature assessment is desirable in light of future clinical translations.

## Introduction

The epigenetic regulation of chromatin structure and gene expression represents a major field of study and intervention for researchers focused on cancer. The class III lysine/histone deacetylases are known as Sirtuins and are represented, in humans, by 7 members (6, 7). Silent information regulator 1 (SIRT1) is the most studied and well characterized among the class III deacetylases and its targets include p53, Ku70, peroxisome proliferator-activated receptor gamma (PPARγ), peroxisome proliferator-activated receptor gamma coactivator 1-alpha (PGC1α), Beclin 1 and β-catenin, among others (8–13). A relevant group of SIRT1 targets is the Forkhead box O (FOXO) family of transcription factors. FOXO3a hyperacetylation mediated by SIRT1 accompanies apoptosis while FOXO4 deacetylation activates this target enhancing its transcriptional and biological activity in the nucleus. FOXO4 activation enhances the cellular defenses against oxidative stress and leads to apoptosis resistance (8, 14, 15). Fibrosis is another aging-related disease involving FOXO1/3, well known modulators of aging and longevity playing a clear inhibitory effects on fibrogenic effector cells and extracellular matrix production (16).

Furthermore, the epigenetic regulation and dysregulation of the hypermethylated in cancer1 (HIC1)/SIRT1/p53 axis is relevant for the development of malignancies (17). SIRT1 is also a target for several miRNAs, small non-coding RNA molecules known to be deregulated in various cancers, whose expression is involved in tumorigenesis (18).

SIRT1, due to its NAD^+^ dependency, is also a metabolic sensor and its deacetylating activity toward regulatory target proteins affects cell metabolism (19).

The focus of this review is the role and involvement of SIRT1 in secretory organ cancers since the information recently gathered on this topic should bear new translational benefits. Correlations between SIRT1 and nuclear receptors, transcription factors, master regulators of gene expression, miRNAs and lncRNAs have been reported. The main glands in the body and the most recent literature are taken into consideration.

## Ovarian cancer

Ovarian cancer is the fifth leading cause of female cancer mortality in the United States (20). This type of cancer is currently treated with a platinum and taxane-based chemotherapy after surgical cytoreduction. However, the rates of progression free survival (PFS) and OS for these patients remain dismal and no benefits seem to arise by the introduction of additional cytotoxic agents (21). Ovarian cancer is usually regarded as a single entity and current treatment protocols for the disease are not subtype specific. Actually, ovarian cancer is a heterogeneous disease that is classified by histopathological examination, including serous, clear cell, endometrioid, and mucinous subtypes. These subtypes develop differently and respond differently to chemotherapy (22).

SIRT1 overexpression is reported by many authors as associated with poor outcome and chemoresistance in ovarian cancer of epithelial origin (23–25). Ovarian cells (both normal and tumoral) express two kinds of estrogen receptors (ER), ERα and ERβ which play different roles in cell proliferation and aggressiveness (4). Specifically, ERα is associated to a poor outcome, while ERβ expression corresponds, on the contrary, to a favorable outcome. ERα levels are closely associated with estrogen-dependent growth and the increased metastatic potential of ovarian epithelial carcinoma (OEC) through the promotion of the epithelial to mesenchymal transition (EMT) (26). ERβ, in contrast, inhibits EMT in presence of 17β-estradiol (E2), thus mediating the opposite effect to ERα (26). SIRT1 is inversely correlated to ERβ mRNA and protein levels, and the specific ERβ activator KB9520 strongly inhibits SIRT1 mRNA expression. These data collectively support the role of SIRT1 as a tumor promoter in OEC (4).

Recently, another association between OEC and SIRT1 was reported (27). These observations are relevant, because OEC patients often acquire resistance to paclitaxel or cisplatin, and the increased expression in *SIRT1* and *TWIST1* (two genes associated with drug resistance) is observed in OVCAR-5 cells that show increased cisplatin resistance, migration and invasion potential (27).

SIRT1 is a downstream target of hypoxia inducible factor 1α (HIF1α) (28). Hypoxia is a condition associated with several types of tumors and, specifically, increased HIF1α expression predicts the poor prognosis of ovarian cancer. Notably it has been demonstrated that SIRT1 expression is induced by hypoxia and that HIF1α silencing indirectly hampers SIRT1 expression. Finally, the NF-κB signaling pathway is involved in hypoxia-induced SIRT1 up-regulation, strengthening the link between this class III lysine deacetylase and ovarian cancer (28).

## Thyroid cancer

**Figure 1:**
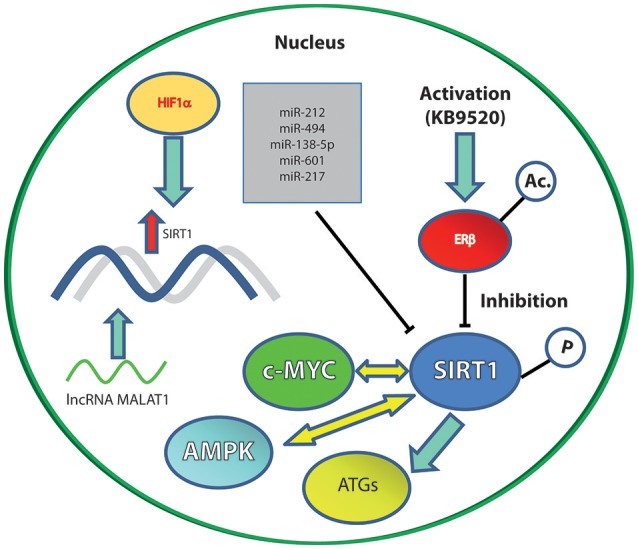
The main SIRT1 molecular interactors (inhibitory or activatory) cited in the text. Solid blue arrows represent an activation; yellow arrows represent an interplay; blunt ended black lines represent an inhibition; red arrow represents upregulation of gene expression.

The incidence of thyroid cancer has increased over the past few decades worldwide. This increase is mainly driven by new cases of papillary thyroid cancer (29). SIRT1 is reported to be oncogenic in thyroid and prostate murine carcinomas initiated by PTEN deficiency (2). The mechanism unveiled by mRNA transcriptional analysis revealed that SIRT1 drives oncogenesis through c-MYC regulation (Figure 1). Two pathways upregulated via SIRT1 overexpression are related to translation and ribosomal biogenesis, which are both controlled by c-MYC. The protein product of the c-MYC oncogene lies at the intersection of a transcriptional network regulating cellular proliferation, replicative potential, cell–cell competition, cell size, differentiation, metabolism, and apoptosis (30).

c-MYC increases in response to mitogenic stimuli, and this is accompanied by SIRT1 upregulation. c-MYC activates SIRT1 by promoting NAMPT transcription and then the NAD-salvage pathway. Furthermore, c-MYC can bind directly to DBC1 (that is a SIRT1 inhibitor) thus preventing SIRT1 blockade (31, 32). On the other hand, SIRT1 activation leads to p53 deacetylation and consequent inactivation, decreasing the levels of PUMA and p21 and eventually counteracting the pro-apoptotic effects of p53. Finally, SIRT1 stabilizes c-MYC via lysine-specific deacetylation and increases its transcriptional activity. Altogether, these data demonstrate a positive feedback loop between c-MYC and SIRT1 (31).

The translational significance of these findings is highlighted by the consistent overexpression of SIRT1 in follicular and papillary thyroid carcinomas, positively correlates with c-MYC protein levels and stabilizes c-MYC (2).

The natural phytochemical resveratrol (RSV) is known to affect mammalian cell metabolism and aging by modulating SIRT1 (33, 34). Furthermore, SIRT1 is also a reported molecular target of RSV in cancer cells of both lymphoid and epithelial origin (35). RSV regulates thyroid stimulating hormone (TSH) secretion via SIRT1 activation. RSV also arrests follicular thyroid and papillary thyroid carcinoma growth by activating the mitogen-activated protein kinase (MAPK) signal transduction pathway and p53 phosphorylation (36, 37).

Among the few studies available, regarding the role of SIRT1 in the thyroid neoplasias, most report involvement as a direct target for miRNAs. miR-212 is a tumor suppressor miRNA that inhibits the proliferation, migration, and invasiveness of thyroid cancer cells. SIRT1 is a direct target of miR-212 and is inversely correlated to miR-212 expression in thyroid cancer tissues (42 samples of human thyroid cancer) (38).

## Pancreatic cancer

Pancreatic cancer (PC) is one of the most deadly tumors in western countries and the 10th most common cancer in the United States. Pancreatic ductal adenocarcinoma is the most common subtype and is recognized as “incurable” due to its aggressive clinical course and resistance to available therapies (39, 40). miR-494 is specifically involved in PC proliferation (41). The overexpression of miR-494 inhibits PC cell proliferation *in vitro* and *in vivo* in addition to inhibiting invasion. The c-MYC/SIRT1 axis is a direct target of miR-494 and there is an inverse correlation between the two. The simultaneous interference of c-MYC and SIRT1 synergistically reduces the expression of these two targets and inhibits PC proliferation, eventually stimulating miR-494 expression (41). The interplay between c-MYC and SIRT1, therefore, plays a relevant role during PC tumorigenesis and should be taken into account when designing strategies aimed at combating this cancer.

Recent studies on the miRNA-mediated regulation of autophagy in PC have been published (42). Since it is known that hypoxia induces autophagy, PC cells were cultured under normoxic and hypoxic conditions. miR-138-5p emerged among the most down-regulated miRNAs in the hypoxia-grown cells. Targets can analysis further revealed that the *SIRT1* gene contains a classical and conserved domain that binds miR-138-5p. Notably, miR-138-5p specifically binds to the 3′-UTR of *SIRT1* and its overexpression leads to SIRT1 protein downregulation (42). Finally, the authors demonstrated that miR-138-5p also counteracts the hypoxia induction in cancer cells through SIRT1 inhibition in tumor xenografts.

Another player that emerged recently is miR-601, which suppresses PC proliferation and migration (43). The target of this miRNA's activity was identified, once more, as SIRT1. This Sirtuin is downregulated by miR-601 and its overexpression reverses the effect of miR-601 on PC cells (43).

miR-217 also directly targets SIRT1, it is inversely correlated to SIRT1 expression and is down-regulated in chronic pancreatitis and PC (44).

The Ex-527 synthetic inhibitor, which inhibits pancreatic cell proliferation *in vitro* and in combination with the first-line drug for this pathology, gemcitabine, is considered one of the most specific SIRT1 inhibitors currently available (45). The same inhibitor was shown to promote PC xenograft tumors in SCID mice and did not synergize with gemcitabine. Therefore, caution should be used when dealing with Ex-527 inhibitor to target SIRT1 *in vivo*.

The role of histone deacetylases (HDACs) is poorly understood in pancreatic neuroendocrine tumors (pNETs). A comprehensive expression pattern of all the five classes (I, IIa, IIb, III, and IV) of HDACs was recently described (3). The gene expression profiles of a total of *n* = 57 patients revealed a significant upregulation of all the HDAC classes in pNETs over controls with increased levels ranging from 1.5- to 7-fold. The expression of several HDACs, including SIRT1, correlates to the G3 stage and, thus, to pNETs tumor grading (3).

## Gastric cancer

Gastric cancer (GC) is among the most prevalent malignancies of the upper gastrointestinal tract. It is a solid aggressive carcinoma where new therapeutic options are warranted (46). Autophagy in GC may have the dual role of promoting the activity of irradiation and anticancer drugs or, on the contrary, may function as a protective mechanism for cancer cells (47–49).

SIRT1 plays a confirmed role in autophagy (50, 51). In GC, it has been demonstrated that SIRT1 deacetylates autophagy-related gene products (ATGs) and a series of histonic and non-histonic targets, eventually interfering with the autophagic process (Figure 1) (52).

GC is among the few human tumors where SIRT1 is downregulated, and in one study, the prognosis of low-SIRT1 expressing GC patients was good (52, 53). However, other authors reported a meta-analysis of SIRT1 expression in gastrointestinal cancer in which the results were heterogeneous (54). The overall results from 15 studies showed an association between worse OS and high SIRT1 expression in gastrointestinal cancer. The subgroup analysis revealed that this association was particularly strong in non-colorectal gastrointestinal cancers, especially GC and hepatocellular carcinoma (54). Therefore, SIRT1 is a promising prognostic factor in GC but not in colorectal tumors.

In contrast with the abovementioned results, a study of the clinicopathological parameters in GC patients was analyzed using the Kaplan-Meier plotter (55). Here, the data concerning the seven human Sirtuins were extrapolated from NCBI GEO databases containing mRNA profiles and the corresponding clinical data of a large number of GC patient samples. The Kaplan-Meier plotter was used to investigate the predictive value of mRNA expression of the Sirtuins for the OS of GC patients. Among a total of 631 available cases for SIRT1 expression, the survival curves suggested that a high expression of SIRT1 mRNA was favorable for OS and that different Sirtuins had diverse correlations with OS. Furthermore, the first progression (FP) was positively correlated with SIRT1 expression: in other words, a longer FP time was associated with a higher SIRT1 level (55).

Opposite evidence was reported from the immunohistochemical (IHC) staining of primary GC tissues. High SIRT1 expression, by IHC, was associated with lymphatic invasion (*p* = 0.028), vessel invasion (*p* = 0.016) and lymph node metastasis (*p* = 0.014) and tended to be associated with more advanced disease stages (56).

Clearly, no consistent data on the exact role of SIRT1 in GC are available at present. The evidence available thus far points to a dual role for SIRT1 (that is to say it may act as a tumor promoter as well as a tumor suppressor), possibly depending on its sub-cellular localization, p53 status, and microenvironmental conditions (57, 58).

## Adrenal glands

The function of SIRT1 in the adrenal glands has been investigated the least. One study reports the researches performed on human adrenal NCI-H295R cells (59) and, in this setting, Sirtuins (including SIRT1) did not seem to be involved in the RSV-mediated lowering of androgen production.

Moreover, steroidogenesis in H295R cells was not affected by Sirtuins, even when SIRT1/3/5 are overexpressed through transgenesis, strengthening the fact that RSV-dependent effects in this setting are SIRT-independent (59).

## Hepatic cancer

Hepatocellular carcinoma (HCC) is a highly malignant tumor with a higher frequency in East Asia than in Europe and North America (60). HCC is the predominant malignancy in the liver and recent evidence shows a role for SIRT1/PGC-1alpha/mitochondrial biosynthesis pathway in protecting the liver against lipid accumulation and mitochondrial dysfunction (61, 62). Senescence induction is a recently proposed strategy with the aim of counteracting HCC (63). Metformin, for instance, induce senescence via the activation of AMP-activated protein kinase (AMPK) when used at low doses. This effect is accompanied by the phosphorylation of SIRT1 as well as p53-acetylation (Figure 1). The prolonged exposure to low doses of metformin leads to human HCC senescence in murine xenografts via modulation of the AMPK-SIRT1 axis (63).

The long non-coding RNA (lncRNA) metastasis associated lung adenocarcinoma transcript 1 (MALAT1) was recently discovered to interfere with SIRT1 activity in hepatocellular cancer (64). MALAT1 is frequently upregulated in several types of cancers and contributes functionally to the development and malignancy of tumor cells (65). MALAT1 is, among lncRNAs, a stable and highly transcribed molecule localized in the nucleus, and it has been reported that, in breast cancer cells expressing ERα, 17β-estradiol treatment negatively regulates MALAT1 transcription, thus inhibiting proliferation, migration and invasion (66). MALAT1 is also regulated during endothelial differentiation in that hypoxia induces its transcription leading to the proliferation of the vasculature (67). In HCC specifically this lncRNA promotes aggressiveness in that releases SIRT1 by sponging miR-204 (64). SIRT1 is a direct downstream target of miR-204 and results inhibited by this interaction. MALAT1 can release the suppression on SIRT1 by base-pairing with miR-204 and leading to its post-transcriptional downregulation (64).

## Conclusions

SIRT1 should be regarded as a multi-faceted enzyme and, as already suggested by several authors, studied in a context-dependent fashion. Secretory organs are no exception to this. Table 1 summarizes the secretory organ tumors in which SIRT1 has been investigated, with the relevant experimental models. The most recent publications are taken into account.

**Table 1:** Cancer models of the secretory organs in which SIRT1 has been studied.

	**Experimental models and specimens**	**References**
Ovarian cancer of epithelial origin	Normal ovaries, endometriosis with/without carcinoma, OvCa (endometrioid; clear cell; mucinous; serous). OvCa cell lines. Serous EOC.	(4, 23–28)
Thyroid carcinoma	Murine thyroid cancers. Thyroid cell lines. Nude mice models. Human normal and tumor thyroid specimens	(2, 37, 38)
Pancreatic cancer	Confirmed human PC and non-tumor pancreatic specimens. PC cell lines. Normal human pancreatic ductal epithelium. PC xenografts. Human chronic pancreatitis specimens	(41–45)
Pancreatic neuroendocrine tumors	Formalin fixed paraffin-embedded human samples	(3)
Gastric cancer	Human primary GC specimens. GC cell lines. Clinical trials concerning gastrointestinal cancers, overall survival and SIRT1. NCBI GEO databases of mRNA profiles. Formalin fixed paraffin-embedded human samples	(52–56)
Hepatic cancer	Primary liver cancer organoids (hepatocellular carcinoma; cholangiocarcinoma; combined HCC/CC; healthy liver). HCC mouse xenografts. HCC cell lines and primary human tissues	(61, 63, 64)

SIRT1 plays a confirmed role in ovarian, thyroid, and pancreatic cancers, while its role in GC is yet to be definitely established. The evidence available in thyroid cancer confirms that c-MYC and SIRT1 are valuable therapeutic targets, encouraging translational researches aimed at the related pathways. The recent meta-analysis spanning 37 selected studies on human solid cancers demonstrates a correlation between higher SIRT1 expression and worse OS in liver and lung cancers (5). These clinical data are consistent with the experimental ones reported for HCC where SIRT1 activation correlates to malignancy through MALAT1, a lncRNA highly expressed in various cancers including HCC (64, 68).

Furthermore, colorectal and GC do not show the correlation between OS and SIRT1 expression (5). SIRT1 overexpression has been reported either positively associated to FP or negatively associated to OS. Contradictory results also concern SIRT1 IHC and mRNA expression and prognosis of GC, suggesting that this class of lysine-deacetylases is not a reliable biomarker for GC.

In contrast, little evidence is available in the literature concerning the role (if any) of SIRT1 in the pituitary gland. In the testis, the role of SIRT1 is reported to be associated with spermatogenesis, fertility, and differentiation, but not testicular cancer (7).

In light of the literature presented here, further basic research is warranted in order to establish possible therapeutic strategies for ovarian, thyroid and pancreatic cancer, where a pro-tumorigenic role for SIRT1 has been established. Particular attention should be paid to the role of miRNAs in regulating SIRT1 levels (miR-212 in thyroid cancer; miR-494, miR-138-5p, miR-601, and miR-217 in PC; miR-204 in HCC). Furthermore, given the growing importance of lncRNAs in cancer pathology, the involvement of MALAT1 should be exploited. For instance, by measuring its levels in HCC biopsies and, possibly, by monitoring its expression during therapy administration or disease progression.

## Author contributions

RF has been studied SIRT1 in cancer and is currently assessing a possible epigenetic regulation for this enzyme. RF conceived, wrote, and formatted the manuscript.

### Conflict of interest statement

The author declares that the research was conducted in the absence of any commercial or financial relationships that could be construed as a potential conflict of interest.
